# Prevalence of molecular markers associated with *Plasmodium falciparum* resistance to chloroquine and sulphadoxine/pyrimethamine in three malaria endemic local areas of Benue State, Nigeria

**DOI:** 10.11604/pamj.2025.50.73.45826

**Published:** 2025-03-13

**Authors:** Amase Nyamngee, Raphael Terlumun Ikpe, Mariam Kehinde Sulaiman

**Affiliations:** 1 Department of Medical Microbiology and Parasitology, Faculty of Basic Clinical Science, College of Health Sciences, University of Ilorin, Ilorin, Nigeria

**Keywords:** Prevalence, molecular markers, *Plasmodium falciparum*, resistance, Nigeria

## Abstract

**Introduction:**

currently, malaria (primarily caused by Plasmodium falciparum) remains prevalent in over 106 countries and is one of the most severe public health problems globally, leading the cause of deaths especially among children and pregnant women particularly in developing countries. This study determined the drug-resistance molecular markers in Plasmodium falciparum infection in three malaria endemic local areas of Benue State, North-central Nigeria between June 2023 and September 2024.

**Methods:**

the conclusive diagnosis of P. falciparum was based on identifying the characteristic asexual stage of the parasite in Giemsa-stained blood smears examined under a compound microscope. The DNA (Deoxyribonucleic acid) extraction from P. falciparum positive blood samples was done using Chelex extraction method. Nested polymerase chain reaction followed by Restriction Fragment Length Polymorphisms (PCR/RFLP) were used for the detection of Plasmodium falciparum Chloroquine resistance transporter (pfcrt), P. falciparum multidrug resistance 1 (pfmdr1), P. falciparum dihydrofolate reductase (pfdhfr) and P. falciparum dihydropteroate synthase (pfdhps). Data were analysed using SPSS Version 24.00 and inferences were drawn for Statistical significance at P<0.05.

**Results:**

the results revealed well-characterized molecular markers of P. falciparum resistance to the 4-aminoquinolines and the antifolate drugs indicating a high prevalence of resistance: 41%, 60%, 51% and 47% of P. falciparum isolates at codons N86Y, K76T, S108N, N51I and A437G respectively.

**Conclusion:**

the prevalence of resistance of isolates to antimalarial drugs was significantly high. Therefore, strategies to reduce multiple-strain infections should be implemented to improve antimalarial drug efficacy and reduce the rate of spread of drug resistance.

## Introduction

Malaria is found in over 106 countries and is one of the most severe public health problems, affecting half of the world's population and being a leading cause of avoidable death, especially among children and pregnant women in many developing countries [1]. Malaria fever originated in Africa and spread throughout the continent as the nomadic lifestyle was abandoned for agriculture which accidentally created favourable habitats for mosquitoes to breed [2]. European colonization and the African slave trade brought malaria to the Americas, where various species of *Anopheles* mosquitoes were available to serve as vectors for transmission [3-5]. Severe malaria is almost exclusively caused by *P. falciparum* infection and usually arises 6-14 days after infection. If untreated, severe malaria can result to coma and death among young children and pregnant women. Splenomegaly, severe headache, cerebral ischemia, hepatomegaly (enlarged liver), hypoglycaemia, and hemoglobinuria with renal failure may occur. Severe malaria can progress extremely rapidly and cause death within hours or days [6,7]. In the most severe cases of the disease, fatality rates can exceed 20%, even with intensive care and treatment. In endemic areas, treatment is often less satisfactory and the overall fatality rate for all cases of malaria can be as high as one in ten. Over the long term, developmental impairments have been documented in children who have suffered episodes of severe malaria [8,9].

The emergence of drug resistance, widespread resistance to available insecticides, wars and massive population movements, difficulties in obtaining sustained funding from donor countries and lack of community participation made the long-term maintenance of the effort to eradicate malaria untenable. The completion of the eradication campaign was eventually abandoned. The goal of most current National Malaria Prevention and Control Programs and most malaria activities conducted in endemic countries is to reduce the number of malaria-related cases and deaths. To reduce malaria transmission to a level where it is no longer a public health problem is the goal of what is called malaria control [10,11]. The genetic complexity of *P. falciparum*, particularly its ability to crossmate and generate mutant variants, makes it a successful pathogen. Genetic variants are involved in pathogenicity and immune responses and have led to the emergence of resistance against virtually all available anti-malaria drugs. Such variants are under strong selective pressure, but analyses of sequence variation in gene segments that are not directly subjected to selection permit the study of interpopulation diversity of the parasite and its evolutionary origin [12,13]. Microsatellites (MS) are molecular markers that can provide valuable information on population structure in malaria parasites and are common in the *P. falciparum* genome. They can be defined as nucleotide repeats of ranges between 2 and 6bp, one MS can be found in every kilo-base genome-wide. MS are highly polymorphic due to the variation of length of these multiple alleles and more neutral than SNPs among parasite isolates. Fortunately, mutations associated with drug resistance in *P. falciparum* provide a unique chance to identify allelic associations because these mutations have occurred within the past 50 years [14]. In Asia and some African countries, microsatellite markers with multiple alleles flanking genes associated with drug resistance such as *dhf* and *pfcrt* were investigated and allowed the typing of specific haplotypes flanking these regions and enabled identification of drug resistance origin as well as tracing spread of these mutations. However, there is no supporting literature of any such study in Nigeria despite the high mortality, morbidity and public health concern about malaria [15,16].

**Objective:** this study determines the prevalence of molecular markers associated with *P. falciparum* resistance to chloroquine and sulphadoxine/pyrimethamine in three malaria endemic local areas of Benue State, Nigeria.

## Methods

**Study design:** a descriptive cross-sectional study was designed for this research work, while subjects were selected randomly among primary school pupils and community dwellers in the 18 communities of three local government areas in Benue State. This study population is known to be adversely rural and underdeveloped with predisposing environmental factors suitable for malaria spread in Benue State.

**Setting:** this study was carried out between June 2023 and September 2024 in 18 local and peri-urban communities of three Local Government Areas in Benue State, North Central Nigeria. This State is located between latitude 7o.40'-10o30'N and longitude 6o.20'-8o.70'E and shares international boundary with Cameroon and six interstate boundaries in Nigeria. The State also lacks basic social amenities, a characteristic of most developing countries.

**Participants:** the target population was school pupils and community members in 18 sites within the three selected local government areas in Benue State.

**Sampling and sample size:** blood specimens were collected from a total of 1,264 participants and screened for malaria parasites based on the high prevalence of malaria as described by Atroosh *et al*. [17]. The details of the study design and sampling technique have been fully described in a previous study [17] and were based on the WHO protocol of 2013 [7], then 100 samples were selected from the confirmed *Plasmodium falciparum* positive cases for molecular detection of mutations in drug targets.

**Data sources/measurement of variables:** a semi-structured questionnaire was used to collect demographic variables, including Age, gender, practice to prevent Malaria, educational level, Indoor residual spraying, History of medication in the last one week (7 days), ownership and use of mosquito net were collected from all the participants in this study. These samples were collected from June 2023 to September 2024 in 18 sites of three local government areas (Guma, Makurdi and Tarkaa) in Benue State.

**PCR for detection of *pfcrt* and *pfmdr*1 Genes:** the detection of mutations responsible for chloroquine resistance was performed by amplifying sequences marking the *Plasmodium falciparum* Chloroquine resistance transporter (*pfcrt*) and *Plasmodium falciparum* multidrug resistance 1 (*pfmdr*1) genes using nested PCR followed by restriction fragment length polymorphism (RFLP) according to previously described procedures [18]. Primers for *pfcrt*(K76T) and *pfmdr*1 (N86Y) primary amplification were Crtp1, Crtp2, Mdr1 and Mdr2 and their sequences. The primary PCR components in a final volume of 15µl was buffer 10x, 25mM Mgcl2, 50mM deoxynucleotide triphosphate (dNTPs), 10µm of each of the primer (Crtp1, Crtp2, Mdr1 and Mdr2), 5u/µl mM deoxynucleotide triphosphate (dNTPs), 10µm of Taq polymerase and 2µl of DNA samples. The cycling protocol for *pfcrt* was as follows: initial denaturation of 94°C for 3 minutes followed by 45 cycles of 94°C for 30 seconds, 60°C for 1 minute and 72°C for 15 minutes. For *pfmdr*1, the cycling protocol was initial denaturation at 95°C for 5 minutes followed by 45 cycles of 95°C for 30 seconds, 45°C for 30 seconds,65°C for 45 seconds and 72°C for 15 minutes.

**Nested PCR and RFLP for *pfcrt* and Mdr 1 mutation-specific detection:** secondary PCR was conducted by using the forward primer Crtd 1 and the reverse primer Crtd2. 2µl of 10x dilution of primary PCR was used in a follow-up, nested, allele-specific PCR amplification to detect the codon for *pfcrt*K76T. The PCR stages for these diagnostic amplifications were initial denaturation at 94°C for 3 minutes followed by 45 cycles at 94°C for 30 seconds, 56°C for 30 seconds and 60°C for 1 minute and a final extension at 72°C for 15 minutes. Purified genomic DNA from *P. falciparum* clones 3D7 (Chloroquine sensitive) and Dd2 (Chloroquine Resistant) were used as positive controls and water and uninfected blood spots on filter paper were used as negative controls.

After amplification, 20µl of the amplicons was incubated overnight at 50°C with mutation-specific restriction enzyme Apo I. In the PCR products, the DNA sequence was cleaved at the wild type (76K) codon site (if present) into two fragments, while the mutant alleles (76T) codon found in chloroquine-resistant *P. falciparum* were not cut. The digested products were separated by electrophoresis in a 2% agarose gel containing ethidium bromide and a DNA was visualised by ultraviolet trans-illumination. Similarly, amplification of codon 86 of the *pfmdr*1 gene was carried out using the following primers: Mdr 1 and Mdr 2 for the primary PCR reactions and Mdr 3 and Mdr 4 for the secondary reactions, after which restrictions with Apo 1 or *Afl*III was done. DNA fragments were compared by size and with the PCR products generated from genomic DNA of the 3D7 and Dd2 strains (used as references for susceptible and resistant genotypes respectively). Thermocycling conditions for *pfmdr*1 secondary reaction were initial denaturation at 95°C for 3 minutes, followed by 25 cycles at 95°C for 30 seconds, 45°C for 30 seconds and 65°C for 45 seconds and a final extension at 72°C for 15 minutes.

**PCR assay for *dhfr* and *dhps* genes:** a nested PCR described by Michael *et al*. [19] was used to amplify fragments of the *dhfr* and *dhps*. The detection of mutations responsible for sulphadoxine and pyrimethamine resistance was performed by amplifying sequences marking the *dhfr* and *dhps* genes using nested PCR followed by restriction fragment length polymorphism. The 15µl *dhfr*/*dhps* final PCR component consisted of buffer 10x, 50mM dNTPs, 25mM MgCl2, 10µm of each of the primer, 5µ/µl of Taq polymerase and 2µl of template DNA. The cycling protocol was as follows: an initial denaturation of 95°C for 5 minutes, 40 cycles of 30 seconds at 95°C, 40 seconds at 52°C, 1 minute at 72°C and elongation at a final cycle of 10 minutes at 72°C. Subsequently, a nested PCR was performed to increase the yields of the specific amplicons, 3µl of the primary PCR product was used in a reaction volume of 15µl with corresponding primers, the PCR mixture was the same as the primary reaction. The cycling condition was previously described [15]. The nested PCR products were confirmed by electrophoresis on a 2% agarose gel stained with ethidium bromide along with a set of controls. The RFLP was performed by incubating the PCR products according to specification with and corresponding restriction enzymes (Alu 1 for *dhfr* S108N/T; Ava II for *dhps* A437G; TSP 5091 for and *dhfr* N51I) and buffers obtained from the bioplolymer factory, Soflinger Str. 100 D-89077 Ulm, Germany. The digested products were visualised by electrophoresis on 2% agarose gels and visualised by ultraviolet trans-illumination.

**Ethical considerations:** ethical clearance was sought and obtained from the University of Ilorin Ethical Review Committee (Ref. UIL/ERC0243/03). A letter of introduction which spelt out the purpose of the research work and benefits to be derived was taken to the Local Government Education Boards, the Education Secretaries as well as Area supervisors and Head Teachers of the various schools.

## Results

**Prevalence of drug resistance markers among confirmed *Plasmodium falciparum* infected populations in malaria endemic local areas of Benue State, Nigeria:** Table 1 presents that the drug resistance marker *pfcrt* (K76T) had the highest percentage of mutant alleles (60%) followed by the *pfdhfr* (N511) with 55% and then *dhps* (A437G) with 47% mutant alleles and the least mutant allele of 16% was observed from the *dhfr*/*dhps* N/108/I51/G437. Among the wide-type alleles, the drug resistance marker *dhfr*/*dhps* N/108/I51/G437 had the highest percentage of 84% and the least percentage of 40% was from *pfcrt* (K76T).

**Table 1:** prevalence of drug resistant markers among confirmed *Plasmodium falciparum* infected population between June 2023 and September 2024 in three malaria endemic local areas of Benue State, Nigeria (N=100)

Drug Resistance Markers	% of Mutant Alleles	% of Wild-type Alleles
Pfcrt (K76T)	60	40
Pfmdr 1 (N86Y)	41	59
Pfdhfr (S108N)	51	49
Pfdhfr (N511)	55	45
PfdhfrN108/I51	29	71
dhps(A437G)	47	53
dhfr/dhps N/108/I51/G437	16	84

**Prevalence of molecular markers associated with *P. falciparum* resistance to chloroquine and sulphadoxine/pyrimethamine by age of the study population in three malaria endemic local areas of Benue State, Nigeria:** Table 2 showed that T76 had the highest mutant alleles of 66% in the age group of 21-30 years and the least percentage of 58.1% in the age group of 1-11 years. The percentage relativity of T76 was closely related across all age groups. In the mutant alleles Y86, the highest percentage of 44.4% was observed in the age group of 21-30 years and the lowest percentage of 40% in the age group of >30 years. The N108 mutant alleles however had the highest percentage of 80% in the age group of >30 years and the least 46.8% in the age group of 1-10 years. The I51 mutant alleles had the highest percentage of 60% also in the same age group of >30 years and the least of 54% in the age group of 1-10 years. For the G437 mutant alleles, the highest percentage of 80% was also from the age group of >30 years and the lowest percentage of 37.5% from the age group of 11-20 years. The variations in the mutant alleles across age groups were statistically significant, P<0.05.

**Table 2:** prevalence of molecular markers associated with *P. falciparum* resistance to chloroquine and sulphadoxine-pyrimethamine by age of the study participants between June 2023 and September 2024 in three malaria endemic local areas of Benue State, Nigeria

Age group	% Examined	No (%) of mutant alleles examined				
(YEARS)	(100)	T76	Y86	N108	I51	G437
**1-10**	62	36(58.1)	25(40.3)	29(46.8)	34(54.8)	30(48.4)
**11-20**	24	15(62.5)	10(41.7)	13(54.2)	13(54.2)	9(37.5)
**21-30**	9	6(66.7)	4(44.4)	5(55.6)	5(55.6)	4(44.4)
**>30**	5	3(60.0)	2(40.0)	4(80.0)	3(60.0)	4(80.0)
**TOTAL**	100	60(60.0)	41(41.0)	51(51.0)	55(55.0)	47(47.0)
**P<0.05**						

**Drug resistance markers by gender of the study populations in the three malaria endemic local areas of Benue State, Nigeria:** the drug resistance marker by gender has been summarized in Table 3 and it shows *pfdhfr* (N108) with the highest percentages of 63.6% in males and *pfcrt* (T76) with the highest percentage of 60.7% in females. Consistently, the males had *pfcrt* (T76) with 59.1%, *pfdhfr* (I51) with 54.5% and *dhfr*/*dhps* N108/I51/G437 with 15.7% while *pfdhfr* (I51) with 55.4%, *pfmdr*1(Y86) with 46.4% and *dhfr*/*dhps* N108/I51/G437 with 16.1% in females but these variations of drug resistance markers between males and females were not statistically significant.

**Table 3:** prevalence of drug-resistance markers by gender of the participants in three malaria endemic local areas of Benue State, Nigeria between June 2023 and September 2024

Drug Resistance markers	Males (44)	FEMALES (56)
	**No.(%) of mutant**	**No.(%) of mutant**
pfcrt (T76)	26(59.1)	34(60.7)
pfmdr1(Y86)	15(34.1)	26(46.4)
pfdhfr(N108)	28(63.6)	23(41.1)
pfdhfr(I51)	24(54.5)	31(55.4)
pfdhfrN108/I51	13(29.5)	16(28.6)
pfdhps(G437)	23(52.3)	24(42.9)
dhfr/dhps N108/I51/G437	7(15.9)	9(16.1)
P>0.05		

**Prevalence of drug resistance markers as distributed among the three malaria endemic local areas of Benue State, Nigeria:** the highest percentage of 76.9% mutant was in Guma and it was the *pfcrt*76 and the least of 37.9% mutant was in Makurdi and it was in the *pfmdr*186. On the wild-type, the highest of 62.1% was in Makurdi and it was in the *pfmdr*186 and the least of 23.1% was still in Makurdi and the *pfcrt*76 group (Table 4).

**Table 4:** prevalence of drug-resistant markers as distributed among three malaria endemic local areas of Benue State, Nigeria between June 2023 and September 2024

Number examined by L.G.As	Pfcrt76		Pfmdr186		Pfdhfr108		pfdhrt51		dhps437	
	Mutant (%)	Wild (%)	Mutant (%)	Wild (%)	Mutant (%)	Wild (%)	Mutant (%)	Wild (%)	Mutant (%)	Wild (%)
Guma (13)	10(76.9)	3(23.1)	6(46.2)	7(53.8)	8(61.5)	5(38.5)	9(69.2)	4(30.8)	8(61.5)	5(38.5)
Tarka (29)	16(55.2)	13(44.8)	13(44.8)	16(55.2)	14(48.3)	15(51.7)	14(48.3)	15(51.7)	13(44.8)	16(55.2)
Makurdi (58)	34(58.6)	24(41.4)	22(37.9)	36(62.1)	29(50.0)	29(50.0)	32(55.2)	26(44.8)	26(44.8)	32(55.2)
**Total**	60(60.0)	40(40.0)	41(41.0)	59(59.0)	51(51.0)	49(49.0)	55(55.0)	45(45.0)	47(47.0)	53(53.0)

## Discussion

### Key results

**Resistance to chloroquine:** one of the factors to be considered in the prophylaxis, treatment, and control of *P. falciparum* malaria is the resistance of parasite strains that may arise against virtually from every drug available overtime. Chloroquine-resistant *P. falciparum* malaria has been a major health problem, particularly in sub-Saharan Africa, leading to changes in treatment policy [20]. According to Gikunju *et al*. [21], chloroquine resistance is been associated in vitro with point mutations in two genes, *pfcrt* and *pfmdr*l, which encodes the *P. falciparum* digestive-vacoule transmembrane proteins and P-glycoprotein-mediated multidrug resistance (Pgh1), respectively. Identification of *pfcrt* as the central determinant of chroloquine-resistant *P. falciparum* malaria provides a molecular marker that can be used for surveillance of resistance to evaluate drug treatment and prophylaxis policies. Analysis of well-characterized molecular markers of *P. falciparum* resistance to the 4-amino-quinolines and the antifolates revealed a high prevalence of resistant genotypes.

The T76 mutation which is associated with chloroquine resistant i.e the substitution of threorine (T76) forlysine(K76) at position 76(K76T) of the amino acid sequence in *pfcrt* which encodes a transporter protein of the *P. falciparum* digestive vacuole was found in 60.0% of samples with *P. falciparum* malaria infection and Y86 mutation (associated with resistance to amodiaquine, mefloquine, halofantrine and lumefantrine) that is the substitution of tyrosine (Y86) for asparagines (N86) at position 86 (N86Y) was found in 41.0%. The high prevalence of resistance to chloroquine observed in this study indicates a persistence of resistance to chloroquine more than 10 years after the change of malaria treatment policy in Nigeria. The result is consistent with the findings from other parts of Nigeria and most regions of high drug resistance [22-24]. This result is however in sharp contrast to findings from Malawi where plasmodium became susceptible to chloroquine after cessation of usage. Also in Tanzania, chloroquine regained its sensitivity after two and a half years of withdrawal of drug pressure.

Chloroquine (CQ) and Sulphadoxine (SP) were replaced with artemisinin in 2005 due to widespread resistance and high-level clinical failure across the country. It is expected that the sensitivity to CQ will be restored about 10 years after withdrawal, as it happened in Malawi and Tanzania. The persistence of CQ resistance witnessed in this study might not be unconnected with poor drug policy in the country, which allows the drug to be freely available for use outside government hospitals. Cross-resistance between CQ and Amodiaquine may also be a contributory factor due to the similarities in their modes of action. Amodiaquine is currently a partner drug with artemisinin in the treatment of malaria in Nigeria. Although this drug remains effective in areas of substantial CQ resistance, the two drugs are chemically related and several clinical and *invitro* reports have shown cross-resistance between CQ and AQ or the active metabolite of AQ [25].

Roux *et al*. [26] observed that *pfcrt*K76 and the *pfmdrl*Y86 alleles are closely associated in chloroquine resistant strains. A similar observation was found in this study as all isolates carrying *pfmdrl*1 mutated allele were also positive for *pfcrt* mutated allele. However, studies comparing the associations of the *pfmdr*1 variant and the *pfcrt*K76 variant have also shown that the impact of the *pfcrt* gene was stronger than that of the *pfmdr* gene [27,28]. It has been suggested that the degree of chloroquine resistance is further modulated by factors linked to genes other than *pfcrt* or *pfmdr* [29]. Consistent with other studies, one can assume that the prevalence of *pfcrt*T76 is a function of the actual chloroquine level and influence of the acquired immunity and natural resistance factors of the host. Furthermore, one can also assume that chloroquine intake contributes essentially to the selection of the *pfcrt*T76 allele.

Regarding *pfmdr*1, the present study showed a predominance of wild type of *pfmdrl*1 N86 (59%). The role of *pfmdrl*1 gene mutations in anti-malarial drug resistance is still controversial. Previous *invivo* studies reported a strong association between *pfmdr*1 86 in the chloroquine-resistant infections [26]. Similarly, a previous study reported a strong association between *pfcrt* K76T but not *pfmdr*1 N86Y mutation *invivo* chloroquine resistance [30]. In contrast, a recent study reported and association with *pfcrt* gene [28]. Moreover, an in vitro study showed that *pfmdr*1 mutations in *P. falciparum* can confer resistance to high levels of chloroquine and that these *pfmdr*1 mutations has an important role in the resistance of *P. falciparum* to mefloquine and quinine [31].

The high prevalence of wild alleles of *pfmdr*1 N86 could be considered as an indicator for low susceptibility of *P. falciparum* isolates to mefloquine, amodiaquine and quinine. On the other hand, previous studies have found increased sensitivity to the anti-malarias mefloquine and artemisinin in *P. falciparum* isolates with mutations in the *pfmdr*1 gene [32]. However, a previous in vivo study found that the treatment failure with mefloquine is significantly associated with increased *pfmdr*1 [33]. This current study did not discover any positive association in the prevalence of mutant alleles in relation to either sex or age of participants, although lower age had lower prevalence of mutant alleles T76 (58.1%) and Y86 (40.3%). However, the differences were not statistically significant, similar to the findings of Okombo *et al*. [13].

**Resistance to sulfadoxine and pyrimethamine:** resistance to sulfadoxine and pyrimethamine was observed in this study among the isolates analysed for resistance markers, *pfdhfr* S108N/N51I and *pfdhps* A437G genes coding for pyrimethamine and sulfadoxine respectively. It has been earlier observed that resistance to pyrimethamine is primarily conferred by a non-synonymous point mutation at codon 108 and is consecutively enhanced by mutations at codons 51 and 59 of the *P. falciparum pfdhfr* gene located on chromosome 4 while point mutations at codon 437 and 540 of the *pfdhps* gene located on chromosome 8 of *P. falciparum* are considered responsible for sulfadoxine resistance [34]. Selection for sulfadoxinepyrimethamine resistance markers in all the study areas remained high even after the replacement of SP as second-line treatment of uncomplicated malaria in 2005. A similar high prevalence of mutant *dhfr* and *dhps* alleles was recorded by Bwire *et al*. [35] in a study conducted at Osogbo, Oyo State, Nigeria. The result is also comparable to that of Dosoo *et al*. [36] and Afutu *et al*. [37].

High prevalence of *dhfr* and *dhps* mutant alleles recorded in this study especially with the presence of double and triple mutant alleles is an indication that there is widespread sulfadoxine/pyrimethamine resistance and this is likely due to continued use of sulfadoxine/pyrimethamine for intermittent preventive therapy in pregnancy (IPTp). Sulfadoxine/pyrimethamine is readily available in both the public and the private sector making its restriction to only IPTp impossible. In the current situation it is unlikely that self-medication with sulfadoxine/pyrimethamine can be prevented especially due to its low cost compared to artemisinin-based combination therapy (ACT), which may also explain the reason for the observed high prevalence of SP resistance markers despite its replacement with ACT. Use of artesunate sulfadoxine/pyrimethamine combination is also another selection factor for sulfadoxine/pyrimethamine resistance markers. The continued use of pyrimethamine has been based partly on the belief that the drug retains activity against the liver stages of *P. falciparum* [35]. Several studies have also shown that although implementation of SP-IPTp does not prevent malaria infection during pregnancy, especially in the presence of high prevalence of SP-resistance markers, there is a significant protection against severe outcomes of pregnancy in malaria, such as low birth weight, maternal and fetal outcomes [38]. This is the reason behind WHO's continued recommendation of SP for IPTp as reported by Mensah *et al*. [39].

**Limitation:** it is expected that a more robust study in scope involving a larger number of participants will be carried out in further studies and in a wider scope of areas or even at regional levels for the prevalence of molecular markers of *Plasmodium falciparum* among all populations. Again as a limitation, this study cannot be generalized.

## Conclusion

It is concluded that the high prevalence of the molecular marker of chloroquine resistance (*pfcrt76*) is suggestive of persistent drug pressure and continuing inefficacy of chloroquine for malaria treatment in the three malaria endemic local areas of Benue State, Nigeria. And that the high prevalence of *dhfr* and *dhps* gene mutations recorded in this study is an indication that an alternative drug would be necessary soon for successful implementation of IPTp in these areas. Therefore, it is essential to continuously monitor the status of the *pfdhfr* and *pfdhps* genes with the goal of better implementation of preventive treatment policies thus we recommend an urgent re-evaluation of the malaria treatment policy in Nigeria and the implementation of effective legislation against the manufacture, importation, sale and use of chloroquine to achieve the desired purpose of the withdrawal. Finally, Regular assessment of the effectiveness of sulfadoxine and pyrimethamine among children and pregnant women is desirable.

### What is known about this topic

- Malaria is still one of the greatest public health problems globally but with particular emphasis in the developing countries;
- High morbidity and mortality rates are being reported among children and pregnant women.

### What this study adds

- This study presents a high prevalence of molecular markers associated with Plasmodium falciparum resistance to chloroquine and sulphadoxine/pyrimethamine in the three malaria endemic local areas studied;
- The study also presents a high prevalence of dhfr and dhps gene mutations across the study population in the study area;
- There are variations in the mutant alleles across age groups in the study area and these variations are statistically significant P<0.05.
